# Item

**Published:** 1900-01

**Authors:** 


### ITEM.

MESSRS. WILLIAM R. WARNER & CO., of Philadelphia, have received a silver medal diploma and blue ribbon, from the National Export Exposition, which recently closed its exhibit at Philadelphia. This is the highest award granted at the exposition, and is further evidence that this well-known house continues to manufacture pharmaceuticals of superiority.

